# The Why, Who, What, How, and When of Patient Engagement in Healthcare Organizations: A Response to Recent Commentaries

**DOI:** 10.15171/ijhpm.2019.42

**Published:** 2019-06-02

**Authors:** Vadim Dukhanin, Matthew DeCamp

**Affiliations:** ^1^Department of Health Policy and Management, Johns Hopkins Bloomberg School of Public Health, Baltimore, MD, USA.; ^2^Johns Hopkins Berman Institute of Bioethics, Baltimore, MD, USA.; ^3^Center for Bioethics and Humanities and Division of General Internal Medicine, University of Colorado, Denver, CO, USA.

We are grateful for the insightful comments received in response to our systematic review of metrics and evaluation tools for patient, public, consumer, and community (P2C2) engagement in healthcare organization- and system-level decision-making.^1^ Given their breadth and depth, it would be impossible to reply to every detail in this brief response. Here we describe areas of broad agreement as well as each comment’s important unique insights, with the hope that doing so will motivate scientific progress in enhancing P2C2 engagement.

All five commentaries emphasize that any evaluation of P2C2 engagement should be guided by its fundamental goals or underlying theory.^2-6^ While we took an inductive approach in this review, we wholeheartedly agree, and our own earlier work reflects that.^7^ However, we bring to attention that our more recent research within a general patient population regarding their perspectives on patient representation. This research indicates that patients are just as concerned about “who” their representatives are and how they can interact with them as they are about the “why” of engagement [V. Dukhanin, S. Feeser, S. A. Berkowitz, M. DeCamp, unpublished materials].^8^ The implications of this deserve further study.

In addition, all five commentaries rightly point out that incorporating studies from other engagement contexts (for example, health research or public health) could have changed our results or informed our discussion of P2C2 engagement in organization-, community-, and system-level healthcare decision-making. We acknowledge this limitation, done for the sake of mythological rigor, and agree regarding the need for those working in engagement to reach across engagement contexts.

Sofaer helpfully reframes our taxonomy into a logic model that is attentive to realistic timelines and the stage of maturation of a particular healthcare organization or engagement strategy.^2^ We find this reframing incredibly helpful and hope that others will also. Sofaer also asks a fundamental question about engagement: Is engagement important in and of itself, or must it be justified by traditional outcomes, such as healthcare quality or costs? An ethical approach to engagement would likely argue that something of value would be lost, if we thought engagement were justified only by these traditional outcomes, but this question requires further critical analysis. Finally, Sofaer reminds us not to confuse failures of practice with failures of theory. We ought not to give up on efforts to purpose meaningful engagement simply because at present P2C2 engagement demonstrates far more of the former.

Like Sofaer, Boivin suggests re-structuring of the taxonomy into structures, processes and outcomes and recommends adapting it to different P2C2 engagement methods (for example, focus groups versus surveys versus a patient-clinician pair co-leading a project).^3^ Boivin also emphasizes the need for greater collaboration among engagement practitioners and the scientists who study it. We agree. In fact, in our experience conducting research with healthcare organizations, the act of research has the potential to improve engagement practice by simultaneously informing engagement initiatives at the organization studied. This is an argument for more research in this area. While perhaps not Boivin’s own view, we refrain from equating P2C2 engagement that lacks evidence of outcomes as purely “tokenistic.” Some process metrics, such as P2C2 representatives’ independence in decision-making or assurance of follow-up commitment to translate recommendations into action, represent real control over engagement and may be just as important as outcomes.

Rahimi and colleagues provided a very useful supplement to our review by evaluating the psychometric properties of the identified P2C2 engagement tools.^4^ This work is exceptionally helpful. Moreover, the invocation to involve broader stakeholders, importantly patient and public representatives, in developing a “consensually agreed structured taxonomy” cannot be overemphasized. One idea for future research would be to involve patients and public representatives in prioritizing elements of the taxonomy to develop a smaller number of core metrics tailored to specific contexts.

Berger asks us to consider the connection between organizational- or system-level engagement and the engagement of individual patients with their clinicians and their healthcare.^5^ Whether one of the goals of P2C2 engagement at the organizational- or system-level should be to affect directly individual-level patient engagement may be debated. If this were a goal, it would certainly have implications for the metrics one would use to evaluate P2C2 engagement, and metrics related to individual-level engagement did not feature prominently in our review. More research is required to address this question.

Danis introduces an important additional dimension to P2C2 engagement evaluation: its timing.^6^ This did not arise in our review, but it is an important addition, and Danis is correct in noting that the results of any evaluation can be affected by when either the evaluation or the engagement itself occurs. In addition, the need to consider the political context of any engagement is critically important; if significant parts of healthcare organizations’ goals are determined by policy requirements, then the potential of P2C2 engagement participants to exert control and influence may be limited. In our view, this is an argument for engaging public in the creation of these policies.

In summary, the vibrant discussion motivated by our systematic review is exactly what it is needed to generate and maintain momentum that will ensure successes of both theory and practice related to incorporating the needs, values, and preferences of patients, the public, consumers, and communities into healthcare delivery.

## Ethical issues

Not applicable.

## Competing interests

Authors declare that they have no competing interests.

## Authors’ contributions

Both authors contributed equally to the design of the manuscript, its drafting and editing for critical intellectual content, and its final approval for publication‏.

## Authors’ affiliations

^1^Department of Health Policy and Management, Johns Hopkins Bloomberg School of Public Health, Baltimore, MD, USA. ^2^Johns Hopkins Berman Institute of Bioethics, Baltimore, MD, USA. ^3^Center for Bioethics and Humanities and Division of General Internal Medicine, University of Colorado, Denver, CO, USA.

## References

[1] Dukhanin V, Topazian R, DeCamp M (2018). Metrics and evaluation tools for patient engagement in healthcare organization- and system-level decision-making: a systematic review. Int J Health Policy Manag.

[2] Sofaer S (2018). Using the taxonomy and the metrics: what to study when and why: Comment on “Metrics and evaluation tools for patient engagement in healthcare organization- and system-level decision-making: a systematic review”. Int J Health Policy Manag.

[3] Boivin A (2018). From craft to reflective art and science: Comment on “Metrics and evaluation tools for patient engagement in healthcare organization- and system-level decision-making: a systematic review”. Int J Health Policy Manag.

[4] Abbasgholizadeh Rahimi S, Zomahoun HTV, Légaré F (2019). Patient engagement and its evaluation tools – current challenges and future directions: Comment on “Metrics and evaluation tools for patient engagement in healthcare organization- and system-level decision-making: a systematic review”. Int J Health Policy Manag.

[5] Danis M (2019). Delving into the details of evaluating public engagement initiatives: Comment on “Metrics and evaluation tools for patient engagement in healthcare organization- and system-level decision-making: a systematic review”. Int J Health Policy Manag.

[6] Berger Z (2018). Metrics of patient, public, consumer, and community engagement in healthcare systems: how should we define engagement, what are we measuring, and does it matter for patient care? Comment on “Metrics and evaluation tools for patient engagement in healthcare organization- and system-level decision-making: a systematic review”. Int J Health Policy Manag.

[7] DeCamp M, Sugarman J, Berkowitz SA (2015). Meaningfully engaging patients in ACO decision making. Am J Accountable Care.

[8] DeCamp M, Dukhanin V, Hebert LC, Himmelrich S, Feeser S, Berkowitz SA. Patients’ views about patient engagement and representation in health care governance. J Healthc Manag. In Press. 10.1097/JHM-D-18-00152 PMC6865800 31498210

